# The specific PKC-α inhibitor chelerythrine blunts costunolide-induced eryptosis

**DOI:** 10.1007/s10495-020-01620-6

**Published:** 2020-07-07

**Authors:** Mehrdad Ghashghaeinia, Pavla Koralkova, Daniela Giustarini, Renata Mojzikova, Birgit Fehrenbacher, Peter Dreischer, Martin Schaller, Ulrich Mrowietz, Antonio Martínez-Ruiz, Thomas Wieder, Vladimir Divoky, Ranieri Rossi, Florian Lang, Martin Köberle

**Affiliations:** 1 Psoriasis-Center, Department of Dermatology, University Medical Center Schleswig-Holstein, Campus Kiel, Rosalind-Franklin-Str. 7, 24105 Kiel, Germany; 2 Physiologisches Institut, Abteilung für Vegetative Und Klinische Physiologie, Eberhard Karls University of Tübingen, 72074 Tübingen, Germany; 3 Department of Biology, Faculty of Medicine and Dentistry, Palacky University Olomouc, Hnevotinska 3, 77515 Olomouc, Czech Republic; 4 Department of Biotechnology, Chemistry and Pharmacy, Laboratory of Pharmacology and Toxicology, University of Siena, Via A Moro 2, 53100 Siena, Italy; 5 Department of Dermatology, Eberhard Karls University of Tübingen, 72074 Tübingen, Germany; 6 Unidad de Investigación, Hospital Santa Cristina, Instituto de Investigación Sanitaria Princesa (IIS-IP), Madrid, Spain; 7 Centro de Investigación Biomédica en Red de Enfermedades Cardiovasculares (CIBERCV), Madrid, Spain; 8 Department of Dermatology and Allergology, School of Medicine, Technical University of Munich, Biedersteinerstr. 29, 80802 München, Germany

**Keywords:** Eryptosis, Costunolide, Chelerythrine, Glutathione, Glucose-6-phosphate dehydrogenase (G6PDH), Phosphatidylserine exposure

## Abstract

Costunolide, a natural sesquiterpene lactone, has multiple pharmacological activities such as neuroprotection or induction of apoptosis and eryptosis. However, the effects of costunolide on pro-survival factors and enzymes in human erythrocytes, e.g. glutathione and glucose-6-phosphate dehydrogenase (G6PDH) respectively, have not been studied yet. Our aim was to determine the mechanisms underlying costunolide-induced eryptosis and to reverse this process. Phosphatidylserine exposure was estimated from annexin-V-binding, cell volume from forward scatter in flow cytometry, and intracellular glutathione [GSH]_i_ from high performance liquid chromatography. The oxidized status of intracellular glutathione and enzyme activities were measured by spectrophotometry. Treatment of erythrocytes with costunolide dose-dependently enhanced the percentage of annexin-V-binding cells, decreased the cell volume, depleted [GSH]_i_ and completely inhibited G6PDH activity. The effects of costunolide on annexin-V-binding and cell volume were significantly reversed by pre-treatment of erythrocytes with the specific PKC-α inhibitor chelerythrine. The latter, however, had no effect on costunolide-induced GSH depletion. Costunolide induces eryptosis, depletes [GSH]_i_ and inactivates G6PDH activity. Furthermore, our study reveals an inhibitory effect of chelerythrine on costunolide-induced eryptosis, indicating a relationship between costunolide and PKC-α. In addition, chelerythrine acts independently of the GSH depletion. Understanding the mechanisms of G6PDH inhibition accompanied by GSH depletion should be useful for development of anti-malarial therapeutic strategies or for synthetic lethality-based approaches to escalate oxidative stress in cancer cells for their sensitization to chemotherapy and radiotherapy.

## Introduction

Costunolide, a sesquiterpene lactone and natural product of plant origin, counteracts tumor growth and metastasis via suppression of STAT3 [1, 2] and NFκB [3] activities (for review see [4]). Costunolide also inhibits differentiation of pro-inflammatory CD4^+^ T cells [5], reduces the activity of the pro-survival enzyme Akt [6] and exhibits anti-bacterial activity [7]. The pharmacokinetic profile of costunolide has been reported [8] and its therapeutic potential is supported by numerous animal studies. Costunolide possesses anti-angiogenic [9, 10] and -osteoarthritic effects [11]. It inhibits pulmonary [12] and hepatic fibrosis [13], induces hair growth [14] and shows strong larvicidal activity [15]. Furthermore, costunolide induces apoptosis, an evolutionary conserved cellular programmed cell death by conjugating with sulfhydryl groups and intracellular thiols, e.g. the reduced form of the tripeptide glutathione (GSH / L-γ-glutamyl-L-cysteineglycine) [16]. This type of non-enzymatic interaction with thiols ultimately leads to complete depletion of both intracellular GSH and its oxidized form (GSSG). This principle was recently demonstrated after treatment of human erythrocytes with parthenolide, dimethyl fumarate and Bay 11–7082, respectively [17, 18]. There is an inverse correlation between intracellular glutathione concentration [GSH]_i_ and the activity of allosterically regulated protein kinase C alpha (PKC-α). The phosphatidylserine (PS)- and calcium (Ca^2+^)-dependent PKC-α, a member of conventional PKCs (cPKCs) family [19, 20] is directly involved in the control of major cellular functions such as neuronal differentiation [21], protein [22] and DNA synthesis [23]. PKC-α enhances survival and proliferation of cancer cells e.g. glioblastoma [24], acute myeloid leukemia cells [25], favours multi-drug resistance [26, 27], cell motility and anti-apoptotic processes [28]. Furthermore, PKC-α-induced Akt activation and their synergistic cross talk acts as a bulwark against stress-induced apoptosis [29]. Interestingly, inhibition of PKC-α commonly triggers apoptosis in nucleated cells [30, 31], while its inhibition protects anucleated human erythrocytes from stress-induced cell death [32, 33], the so-called eryptosis [34]. Eryptosis is triggered by a plenty of xenobiotics [35] and inhibited by several other xenobiotics or by endogenous molecules, including nitric oxide [36], GSH [37] and erythropoietin [38, 39]. Due to a wide range of biological activities, costunolide has been extensively studied over the past 20 years. Our laboratory previously published that costunolide induces eryptosis [40]. The present study explored the effects of costunolide on pro-survival factors and enzymes in human erythrocytes, e.g. GSH and glucose-6-phosphate dehydrogenase (G6PDH), and analysed the effects of the specific conventional PKC-α and -β inhibitor chelerythrine [41–44] on costunolide-induced eryptosis and the GSH synthesis machinery. We have provided new insights into the mechanism of costunolide action that broaden its therapeutic potential. Inhibition of G6PDH and GSH metabolism using costunolide may offer a promising therapeutic strategy to eliminate malaria-infected erythrocytes [45]. Moreover, increase cancer cells’ oxidative stress renders them vulnerable to therapeutic interventions using chemotherapy and radiotherapy [46]. Therefore, understanding the mechanisms of G6PDH inhibition accompanied by GSH depletion should be also useful for exploiting synthetic lethal interactions for targeted cancer therapy.

## Results

### Impact of costunolide on eryptosis, cell shrinkage and hemolysis

**Fig. 1:**
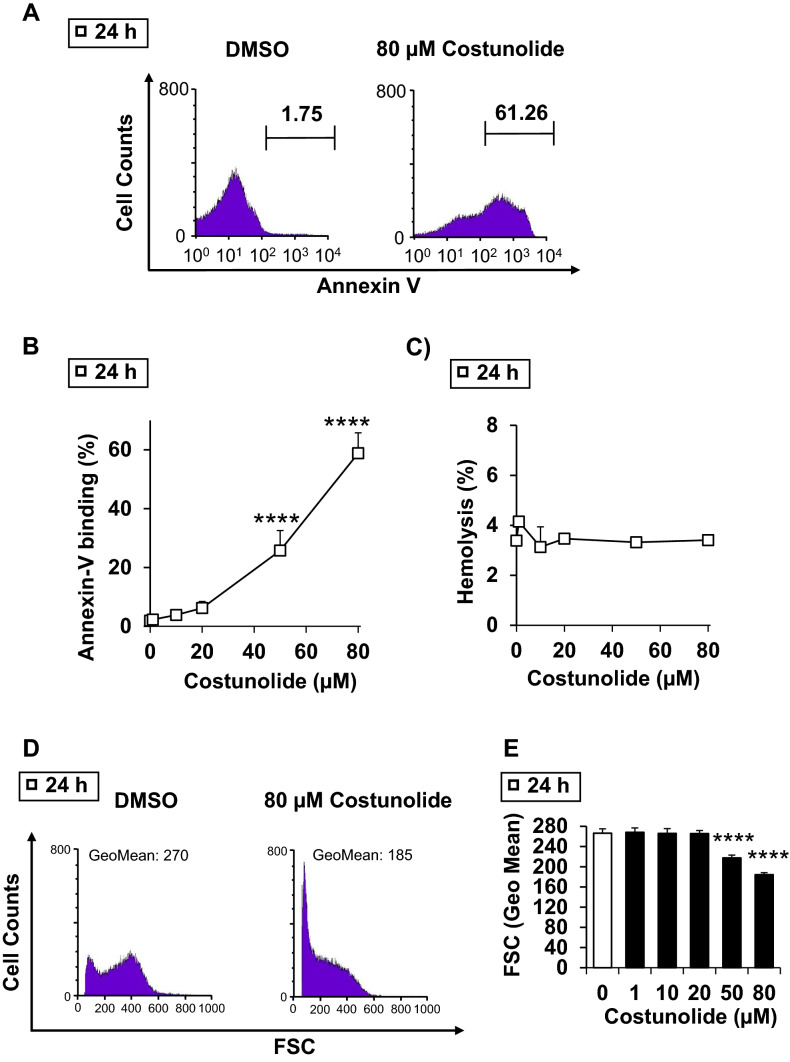
Costunolide-induced eryptosis and cell shrinkage in mature human erythrocytes. Original histograms of annexin V-binding (**a**), concentration-dependent increase of annexin V-binding cells (**b**), concentration-dependent effect on hemolysis (**c**), original histograms of forward scatter (FCS) (**d**), and concentration-dependent decrease of forward scatter (FCS) (**e**) after treatment of human erythrocytes for 24 h with costunolide are shown. Number of independent experiments: n = 3. Differences of the means were considered to be statistically significant when the calculated p value was less than 0.05 (*p < 0.05, **p < 0.01, ***p < 0.001, ****p < 0.0001)

**Fig. 2:**
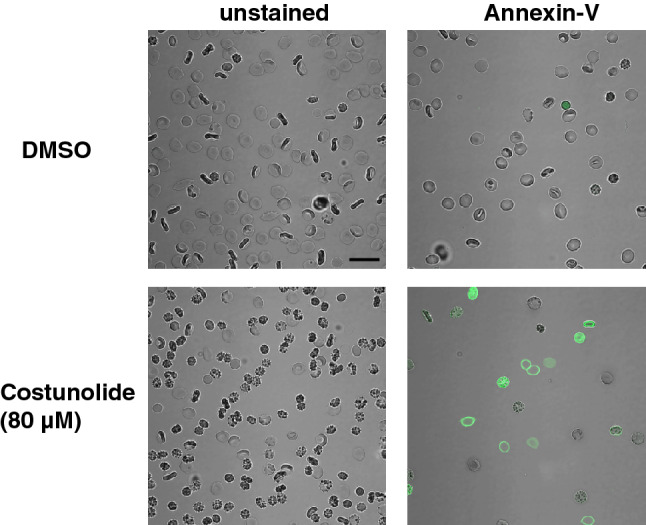
Costunolide-induced annexin V-binding. Human erythrocytes were either treated with DMSO (upper panel) or 80 µM costunolide (lower panel) for 24 h. Fluorescence images of annexin V-stained (right panel) or unstained erythrocytes (left panel) are shown. Scalebar: 20 µm

Human erythrocytes were treated with various physiological concentrations of costunolide. After 24 h, its influence on phosphatidylserine (PS) exposure, cell shrinkage and hemolysis was examined. Increasing concentrations of costunolide paralleled the rates of PS exposure (Fig. 1a, b) and cell shrinkage (Fig. 1e, d) without affecting the hemolysis (Fig. 1c). Fluorescence microscopy confirmed the appearance of shrunken, PS-positive erythrocytes after costunolide treatment (Fig. 2).

### Impact of costunolide on glucose-6-phosphate dehydrogenase (G6PDH) activity and glutathione level

**Fig. 3:**
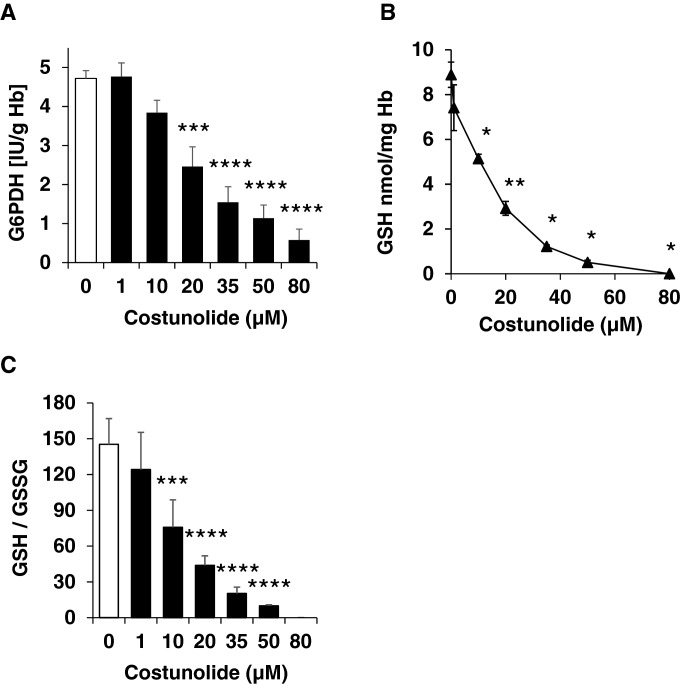
Effects of costunolide on glucose-6-phosphate dehydrogenase (G6PDH) activity, GSH and GSSG levels. Concentration-dependent inhibition of G6PDH activity (**a**), depletion of GSH (**b**), reduction of the GSH/GSSG ratio (**c**) after 24 h treatment of human erythrocytes with costunolide are shown. Number of independent experiments: n = 6. Differences of the means were considered to be statistically significant when the calculated p value was less than 0.05 (*p < 0.05, **p < 0.01, ***p < 0.001, ****p < 0.0001)

The next step was to investigate if costunolide-induced eryptosis is caused by impairment of the redox balance of human erythrocytes. For this, the influence of costunolide on the activity of the pro-survival enzyme G6PDH as well as on glutathione levels (GSH, GSSG) was studied. Indeed, costunolide was able to completely inhibit G6PDH activity (Fig. 3a) and to deplete the reduced form of glutathion (GSH) thereby decreasing the GSH/GSSG ratio in a concentration-dependent manner (Fig. 3b, c). The physiological concentration of GSSG in erythrocytes is very low compared to the GSH level. During costunolide treatment, the GSSG level remained nearly constant, only a slight decrease was observed at a concentration of 50 µM (Table 1). The NADPH-producing enzymes G6PDH and 6-phosphogluconate dehydrogenase (6-PGD) belong to the pentose phosphate pathway [47]. Interestingly, costunolide did not affect the activities of 6-PGD and glutathione reductase (data not shown).

**Table 1:** GSH and GSSG levels after 24 h of costunolide treatment

	DMSO [v/v]	Costunolide [µM]					
	0.2%	1	10	20	35	50	80
GSH [nmol/mg Hb]	8.89	7.41	5.14	2.92	1.22	0.50	0.00
GSSG [nmol/mg Hb]	0.07	0.06	0.08	0.07	0.07	0.05	0.07

### Inhibitory effect of chelerythrine on costunolide-induced eryptosis and cell shrinkage

**Fig. 4:**
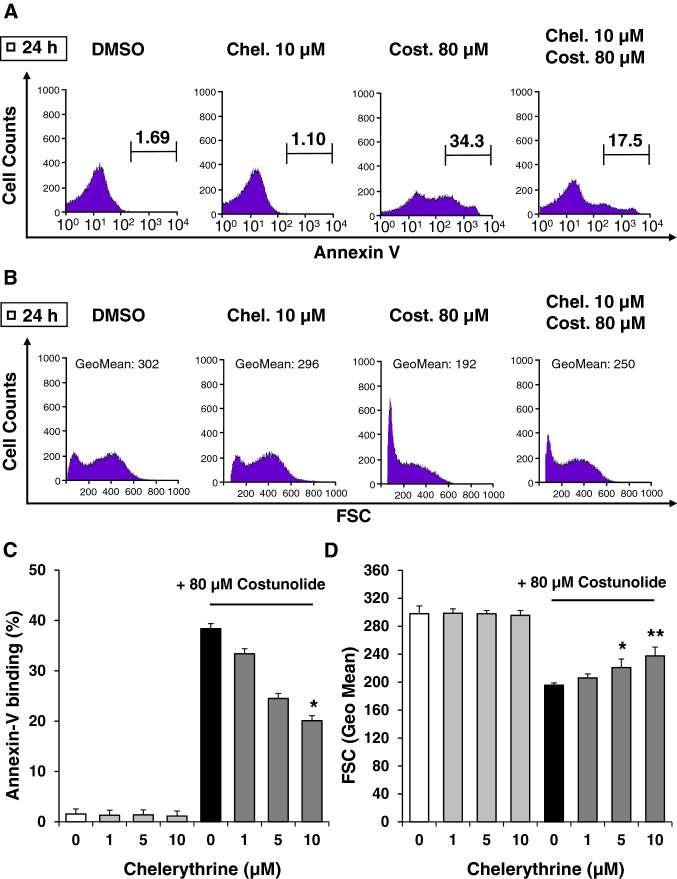
Chelerythrine blunts costunolide-induced annexin V-binding and erythrocyte shrinkage. Human erythrocytes were preincubated with the PKC-α inhibitor chelerythrine and then treated with 80 µM costunolide for 24 h. Original histograms of annexin V-binding (**a**), original histograms of forward scatter (FCS) (**b**), concentration-dependent decrease of costunolide-induced annexin V-binding (**c**), and concentration-dependent increase of forward scatter (FCS) of costunolide-shrunken erythrocytes (**d**) after treatment of human erythrocytes as described above are shown. Number of independent experiments: n = 3. Differences of the means were considered to be statistically significant when the calculated p value was less than 0.05 (*p < 0.05, **p < 0.01, ***p < 0.001, ****p < 0.0001). Chel: chelerythrine; Cos: costunolide

**Fig. 5:**
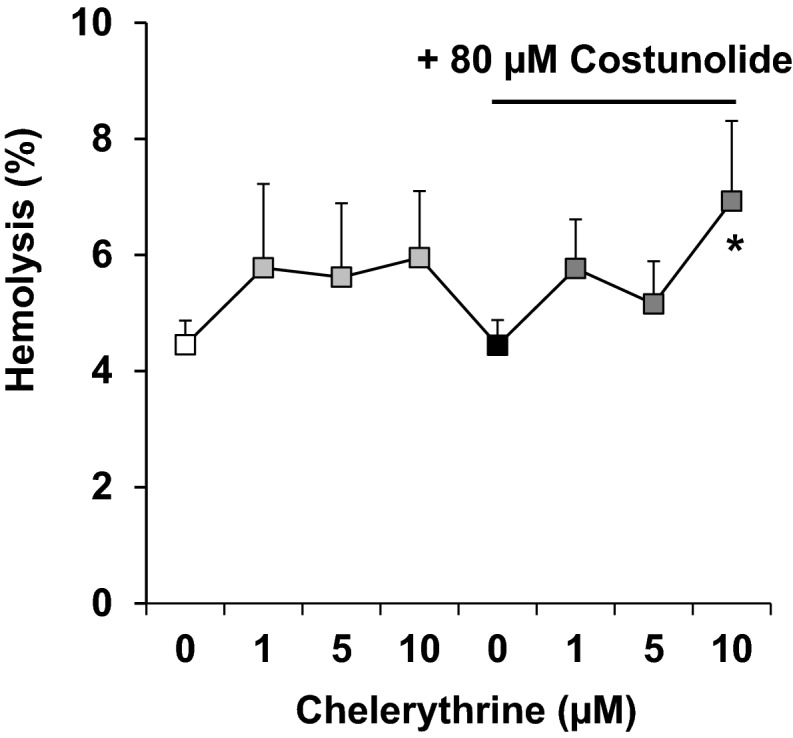
Effect of costunolide and chelerythrine on hemolysis. Human erythrocytes were preincubated with the PKC-α inhibitor chelerythrine and then treated with 80 µM costunolide for 24 h. The concentration-dependent effect of chelerythrine on hemolysis in the absence (left) or presence of costunolide (right) is shown. Number of independent experiments: n = 3. Differences of the means were considered to be statistically significant when the calculated p value was less than 0.05 (*p < 0.05)

We then examined whether chelerythrine, a natural benzophenanthridine alkaloid, has the potential to inhibit costunolide-induced eryptosis and cell shrinkage. To achieve this aim, human erythrocytes were first treated for 2 h with various concentrations of chelerythrine (1 to 10 µM) followed by addition of the highest costunolide concentration (80 µM). In fact, chelerythrine was able to partially inhibit costunolide-induced phosphatidylserine exposure (Fig. 4a, c) and cell shrinkage (Fig. 4b, d). In comparision to DMSO-treated erythrocytes, a combination of the highest costunolide and chelerythrine concentrations induced slight hemolysis (4.45% vs. 6.93%) (Fig. 5).

### Chelerythrine does not impair the glutathione synthesis machinery of mature human erythrocytes

**Fig. 6:**
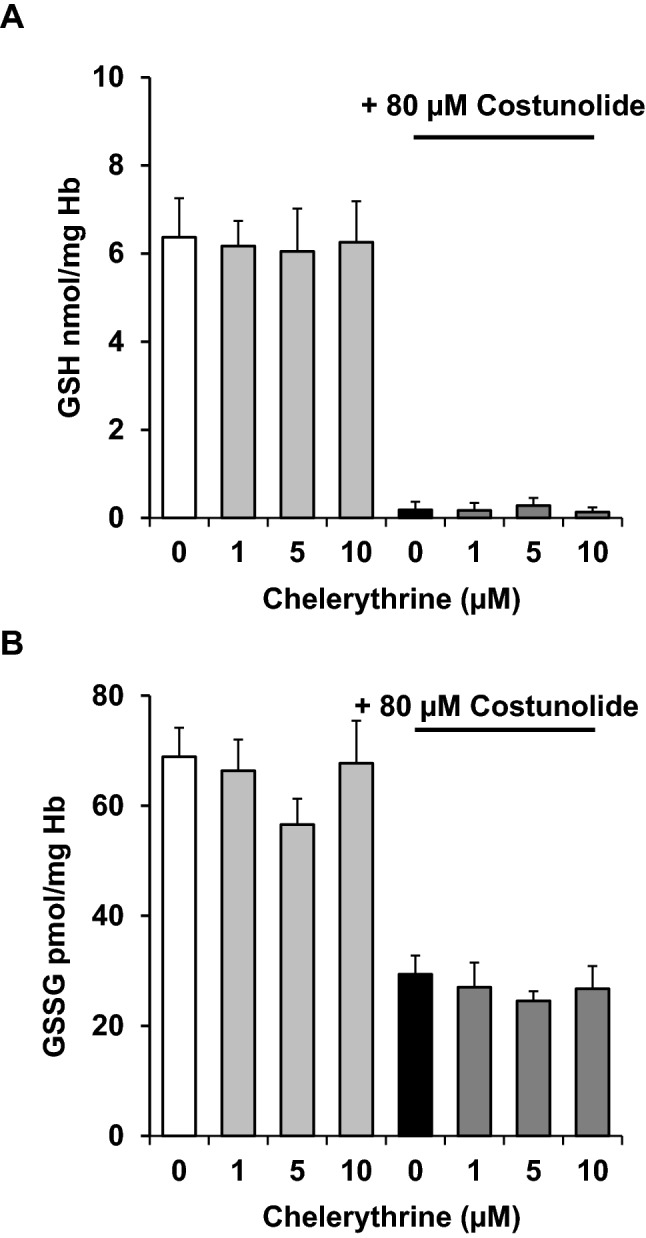
Chelerythrine does not affect costunolide-induced GSH- or GSSG-depletion. Human erythrocytes were preincubated with the PKC-α inhibitor chelerythrine and then treated with 80 µM costunolide for 24 h. Concentration-dependent effects of chelerythrine on GSH (**a**) or GSSG levels (**b**) after treatment of human erythrocytes in the absence (left) or presence of costunolide (right) are shown. Number of independent tests: n = 5. Note that chelerythrine did not significantly affect the GSH (**a**) or GSSG (**b**) levels

Finally, we investigated whether chelerythrine interferes with costunolide-induced GSH depletion. As shown in Fig. 6a, b, chelerythrine did neither reverse the costunolide-induced GSH depletion nor the costunolide-induced slight decrease of the GSSG level. Thus, in our experimental setup chelerythrine seems to act downstream of the glutathione cascade and has no influence on GSH levels.

## Discussion

The present study shows that costunolide triggers eryptosis with cell shrinkage (Figs. 1 and 2). Eryptosis-inducing substances may dose-dependently cause both cell swelling and cell shrinkage [36]. Costunolide induces a sustained influx of Ca^2+^ ions [40], which activates the Gardos channel, a Ca^2+^-sensitive and K^+^-selective channel, by which cellular K^+^ ions leave the cell followed by chloride ions and water, thus leading to erythrocyte shrinkage [48]. Volume-sensitive transport pathways are regulated by kinase(s) and phosphatase(s) activities [49, 50].

We further investigated if costunolide-induced eryptosis was caused by impairment of the redox balance of human erythrocytes. Costunolide was indeed able to inhibit the activity of the pro-survival enzyme G6PDH completely (Fig. 3a) and deplete GSH in a concentration-dependent manner (Fig. 3b and c). The GSSG concentration, however, remained at a very low level independent of the costunolide concentration used (Fig. 3d). This type of GSH depletion that is completely decoupled from GSSG formation has already been demonstrated with other compounds, such as Bay 11-7082, parthenolide and dimethyl fumarate [17].

The linear tripeptide glutathione (GSH), a major non-protein thiol plays a vital role in both prokaryotic [51] and eukaryotic cells [52, 53]. Here, we focus on the vital functions of GSH in mature human erythrocytes. GSH with its turnover time of about 4–6 days and its total intracellular concentration of 3 mM [54, 55], is a linchpin of cellular defences protecting cells from biotic and abiotic stresses. GSH is involved in detoxification mechanisms [56, 57], destruction of free radicals [58, 59], protection of hemoglobin [60, 61], post-translational modification of thiol proteins, the so-called glutathionylation [62], ascorbate recycling and defences against oxidant damage of cell membranes [63, 64]. In addition, GSH depletion is associated with the induction of eryptosis [17, 45]. It is to note that (a) ATP-dependent GSH synthesis is the result of two concerted enzymatic step [47, 65, 66], (b) the capacity of erythrocytes to synthesize GSH exceeds the rate of GSH turnover by 150-fold [67], (c) erythrocytes are permeable to oxidized glutathione (GSSG) whose efflux is an active unidirectional process [68] and (d) GSSG is rapidly recycled to GSH by the NADPH-dependent enzyme glutathione reductase [69–71]. NADPH, as an electron donor reductant, is mainly provided via the irreversible dehydrogenase/decarboxylase system of the oxidative branch of the pentose phosphate pathway by the enzymes G6PDH and 6-phosphogluconolactonase [47]. Thus, it is plausible that G6PDH inhibition should lead to a depletion of NADPH, diminishing the activity of glutathione reductase. This would deplete GSH while excess GSSG is transported outside the cell. Numerous works have been published on NADPH production and its absolute necessity for fatty acid biosynthesis [47, 72, 73] and folate metabolism [74, 75] in mammals. Based on the clinical relevance of GSH, NADPH and G6PDH in the regulation of several human diseases, the modulation of GSH levels and G6PDH acitivity by costunolide is a therapeutic approach to influence the course of various diseases.

In the next step, we examined whether chelerythrine, a natural benzophenanthridine alkaloid and specific inhibitor of conventional protein kinase C (cPKC) can inhibit costunolide-induced eryptosis and cell shrinkage. To achieve this aim, human erythrocytes were first treated for two hours with various concentrations of chelerythrine (1 to 10 µM) and then the highest concentration of costunolide (80 µM) was added. In fact, chelerythrine was able to inhibit costunolide-induced eryptosis and cell shrinkage (Fig. 4). Human erythrocytes possess four isoforms of PKCs: alpha, zeta, mu and iota, of which only the subtype PKC-α translocates to the plasma membrane in order to perform biological activities there [76]; i.e. induction of eryptosis [32, 33]. Furthermore, PMA-stimulated PKC-α activation and the resulting Ca^2+^ influx and cell shrinkage are significantly inhibited by chelerythrine [42]. It is to note that in comparision to DMSO-treated erythrocytes, a combination of the highest costunolide and chelerythrine concentrations induced slight hemolysis (4.45% vs. 6.93%) (Fig. 5).

**Fig. 7:**
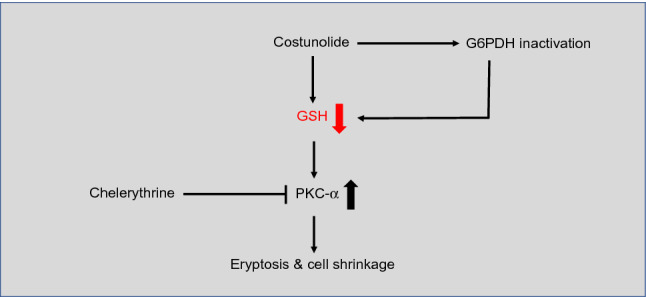
Schematic illustration of inhibition of costunolide-induced eryptosis and cell shrinkage by PKC-α inhibitor chelerythrine

Finally, we investigated whether chelerythrine can influence the costunolide-caused GSH depletion in any way. This was not the case, showing that chelerythrine likely acts downstream of GSH depletion in mature human erythrocytes (Fig. 6). The overall results of this study are summarized in Fig. 7.

In conclusion, costunolide-induced eryptosis is due to its ability to deplete GSH and inhibit the activity of the pro-survival enzyme G6PDH. Chelerythrine as a specific inhibitor of conventional PKC-α and -β isoforms is able to inhibit costunolide-induced eryptosis and cell shrinkage. This work therefore functionally links costunolide and PKC-α. Furthermore, we show that chelerythrine does not influence GSH depletion indicating that the effect of PKC-α in human erythrocytes may occur downstream of GSH depletion. However, a direct effect of costunolide on PKC-α can not be excluded; the exact mechanism how chelerythrine reverses costunolide-induced eryptosis and cell shrinkage requires further studies. Both the induction of eryptosis and its inhibition are of major clinical relevance. The former can be used to combat malaria or to eliminate cancerous erythroid cells through synthetic lethal interactions and the latter to treat anemia-related diseases.

## Materials and methods

### Study design

Single treatment: Erythrocytes were treated with costunolide for 24 h. Double treatments: Erythrocytes were pre-incubated with chelerythrine for 2 h, followed by addition of costunolide for further 24 h. Thus, the incubation time for chelerythrine and costunolide was 26 and 24 h, respectively. In single (0.2% v/v DMSO) and double treatments (0.4% v/v DMSO), DMSO-treated cells served as negative controls. The treatment procedures and incubation were performed under aseptic conditions and all standardized requirements were met. Erythrocyte samples were incubated at 37 °C and gently mixed by inverting or vortexing the tubes (50 ml or 5 ml) several times during the incubation period.

### Erythrocytes

Highly purified erythrocyte suspensions from healthy volunteers with white blood cell or thrombocyte contaminations below 0.1% [77] were provided by the blood bank of the University of Tübingen. Aliquots of the individual erythrocyte concentrates were either used directly at 0.6% hematocrit (Hct) or stored at 4 °C for up to one week. The study was approved by the ethics committee of the University of Tübingen (184/2003 V), the study was performed in agreement with the declaration of Helsinki, and volunteers gave written consent.

### Incubation time

In our experimental setup the decisive factor was the concentration and incubation time at which costunolide was able to achieve complete G6PDH inhibition and GSH depletion. It turned out that a 24-h incubation of the erythrocytes was the most suitable.

### Solutions

Experiments analysing annexing V binding and cell volume (0.6% Hct) were carried out in Ringer solution. Staining of erythrocytes with annexin-V-FLUOS was performed in annexin binding buffer. Ringer solution was composed of (in mM): 125 NaCl, 5 KCl, 1.2 MgSO_4_, 32 N-2-hydroxyethyl-piperazine-N´-ethanesulfonic acid (HEPES)/NaOH (pH 7.4), 5 glucose, and 1 CaCl_2_. Annexin-binding buffer contained (in mM): 125 NaCl, 10 HEPES/NaOH (pH 7.4), and 5 CaCl_2_. Ringer solution and annexin binding buffer were sterile filtered. For this purpose the Setrile-Vaccum-Filtration-System of Millipore was used (pore size of the filter: 0.22 µm).

### Chemicals

1 mg chelerythrine was dissolved in 520 µl and 5 mg costunolide in 538 µl DMSO to achieve 5 mM and 40 mM stock solutions, respectively. These stocks were subsequently aliquoted and stored at − 20 °C for up to one month. Annexin-V-FLUOS was also aliquoted and stored at − 20 °C for several months. Chelerythrine, costunolide, DMSO, annexin-V-FLUOS and N-Ethylmaleimide (NEM) were purchased from Sigma (Taufkirchen, Germany).

### Annexin-V-FLUOS working concentration

On the day of the measurements, the required annexin-V-FLUOS was diluted 1:33 in annexin binding buffer. 48 µl of this solution were taken for staining one sample containing 3 × 10^6^ erythrocytes (for more details see flow cytometry).

### Flow cytometry

At the end of the incubation period, 0.1 ml erythrocytes (3 × 10^6^) were added to 500 µl annexin wash buffer, mixed thoroughly and pelleted by centrifuging. Erythrocyte pellets were gently vortexed to obtain a homogeneous cell suspension. To detect the exposure of phosphatidylserine (PS) on the outer leaflet of the plasma membrane (a measure of the percentage of eryptotic cells), erythrocytes were stained with 48 µl of diluted annexin-V-FLUOS and carefully vortexed. After an incubation period of 20 min in the dark at room temperature, 200 µl annexin binding buffer was added to each sample, thoroughly vortexed to obtain single cell suspensions, and analyzed by flow cytometry on a FACS Calibur (Becton Dickinson, Heidelberg, Germany) as described. Binding of annexin-V-FLUOS (eryptosis) was measured in the FL1-channel. Erythrocyte volume was determined by analyzing the forward scatter (FSC). To this end, corresponding erythrocytes suspensions were immediately analysed by flow cytometry.

### Hemolysis measurement

After 24 h treatment of erythrocytes (0.6% Hct) with costunolide and/or chelerythrine, hemolysis was determined. 600 µl cell suspension from each condition containing 3.6 × 10^6^ erythrocytes were centrifuged for 8 min at 420×*g*, 4 °C, and the supernatants were harvested. As a measure of hemolysis, the hemoglobin (Hb) concentration of the supernatants was determined photometrically at 405 nm. The absorption of the supernatant of DMSO-treated erythrocytes lysed in 600 µl distilled water was defined as 100%, and a standard curve was established by a serial dilution.

### Fluorescence microscopy

After incubation with costunolide, erythrocytes were stained with Annexin-FLUOS as described above. 10 µl of the suspension were applied to a slide and covered with a coverglass. Finally, the cells were analyzed on a LSM 800 microscope (Zeiss Oberkochen, Germany) operated under the Zen software (Version 2.3).

### Intracellular GSH and GSSG analysis

For single or double treatments, pure erythrocytes (0.6% Hct) were suspended in 30 ml Ringer solution and treated with varying concentrations of costunolide (1–80 µM] or different concentrations of chelerythrine (1–10 µM) in combination with the highest costunolide concentration (80 µM). DMSO-treated erythrocytes served as a negative control. After the incubation time (24 h), 58 µl from a 310 mM NEM stock was given to each sample, gently mixed for 1 min and then centrifuged at 10 °C, 228×*g*. Supernatants were removed and the cell pellets were stored at − 20 °C until analyses. GSH and GSSG were measured on the clear supernatant obtained by treatment of 0.1 ml erythrocytes with 0.12 ml 15% (w/v) trichloroacetic acid. For GSH analysis one aliquot (0.05 ml) of supernatant was loaded onto HPLC and the GS-NEM conjugate was revealed by a diode-array detector at 265 nm wavelength [78]. GSSG was measured at the spectrophotometer by the GSH recycling method with slight modifications [79]. One aliquot of erythrocytes (10 μl) was hemolyzed by a 1:200 dilution with H_2_O for hemoglobin determination [80]. The HPLC analyses were carried out by an Agilent series 1100 instrument. The spectrophotometric analyses were performed by a Jasco V-530 instrument.

### Determination of G6PDH activity

Pure erythrocytes (0.6% Hct) were incubated in 20 ml Ringer solution and treated with DMSO or different concentrations of costunolide. 24 h later, cell suspensions were centrifuged at 10 °C, 228×*g*. Supernatants were removed and the cell pellets were stored at − 80 °C until analyses. G6PDH activity was measured in erythrocytes lysates according to methods recommended by the International Committee for Standardization in Hematology [81]. The activity was expressed in units per grams of hemoglobin (IU/g Hb).

### Statistical analysis

Data are presented as the mean values ± SEM of at least 3 independent experiments with different blood samples. A total of 18 different blood samples were used in this study. One-way ANOVA with Dunnet’s post test was used for statistical comparisons of treated samples with controls. Differences of the means were considered to be statistically significant when the calculated p value was less than 0.05 (*P < 0.05, **P < 0.01, ***P < 0.001, ****P < 0.0001).

## Data Availability

Raw data can be provided upon request.
